# Kansas City Cardiomyopathy Questionnaire Utility in Prediction of 30-Day Readmission Rate in Patients with Chronic Heart Failure

**DOI:** 10.1155/2016/4571201

**Published:** 2016-10-30

**Authors:** Shengchuan Dai, Manoucher Manoucheri, Junhong Gui, Xiang Zhu, Divyanshu Malhotra, Shenjing Li, Jason D'souza, Fnu Virkram, Aditya Chada, Haibing Jiang

**Affiliations:** ^1^Internal Medicine Residency Program, Department of Medicine, Florida Hospital Orlando, Orlando, FL, USA; ^2^Division of Cardiology, University of Illinois at Chicago, Chicago, IL, USA; ^3^Center for Interventional Endoscopy, Florida Hospital Orlando, Orlando, FL, USA; ^4^Division of Cardiology, University of South Dakota, Vermillion, SD, USA

## Abstract

*Background*. Heart failure (HF) is one of the most common diagnoses associated with hospital readmission. We designed this prospective study to evaluate whether Kansas City Cardiomyopathy Questionnaire (KCCQ) score is associated with 30-day readmission in patients hospitalized with decompensated HF.* Methods and Results*. We enrolled 240 patients who met the study criteria. Forty-eight (20%) patients were readmitted for decompensated HF within thirty days of hospital discharge, and 192 (80%) patients were not readmitted. Compared to readmitted patients, nonreadmitted patients had a higher average KCCQ score (40.8 versus 32.6,* P* = 0.019) before discharge. Multivariate analyses showed that a high KCCQ score was associated with low HF readmission rate (adjusted OR = 0.566,* P* = 0.022). The* c*-statistic for the base model (age + gender) was 0.617. The combination of home medication and lab tests on the base model resulted in an integrated discrimination improvement (IDI) increase of 3.9%. On that basis, the KCQQ further increased IDI of 2.7%.* Conclusions*. The KCCQ score determined before hospital discharge was significantly associated with 30-day readmission rate in patients with HF, which may provide a clinically useful measure and could significantly improve readmission prediction reliability when combined with other clinical components.

## 1. Introduction

It is estimated that heart failure (HF) affects over 5.7 million Americans with 870,000 new cases diagnosed each year. The predicted prevalence is estimated to increase 46% from 2012 to 2030, resulting in over 8 million individuals suffering with HF [1]. The cost of caring for HF patients was about $30.7 billion in 2012 and is estimated to increase by 127% to $69.7 billion by 2030 [1]. Despite advances in understanding and treatment, the mortality rate of HF remains extremely high with 50% of patients dying within 5 years of initial diagnosis [2].

Readmission of HF after hospitalization is common, and unfortunately many of these readmissions are predictable and possibly preventable [2, 3]. Although new data showed reduction in Medicare hospital readmission rates [4], HF is still one of the most common diagnoses associated with 30-day readmission; an analysis of 2007 to 2009 Medicare claims-based data showed that 24.8 percent of beneficiaries admitted with HF were readmitted within 30 days and 35.2 percent of those readmissions were for HF [5]. These concerning statistics paved the way for a stronger focus on tools to predict and prevent such readmissions.

The Kansas City Cardiomyopathy Questionnaire (KCCQ) was a tool initially designed to provide a better description of health-related quality of life in patients with HF [6]. This questionnaire identified the following clinically relevant domains: physical limitations (question 1), symptoms (frequency [questions 3, 5, 7, and 9], severity [questions 4, 6, and 8], and change over time [question 2]), self-efficacy and knowledge (questions 11, 12), social interference (question 16), and health-related quality of life (questions 13–15) [6]. Previous studies have shown that KCCQ score correlated with survival and hospitalization in patients with HF [7] and was an independent predictor of poor prognosis in this patient population [8]. In addition, KCCQ score measured 1 week after hospital discharge independently predicted one-year survival free of cardiovascular readmission [9]. More recently, KCCQ has also been studied during acute HF hospitalization and demonstrated sensitivity to acute changes, but score changes during hospitalization did not predict short-term readmission [10], although it was a relatively small study, with a sample size of only 52 patients, and it did not investigate the relationship between KCCQ score and HF readmission. Therefore, whether KCCQ score can be used to predict the short-term readmission has yet to be completely evaluated.

To address these gaps in knowledge and explore the feasibility of using the KCCQ score to predict the short-term HF readmission, we designed and conducted this prospective study.

## 2. Methods

The study was approved by the Florida Hospital Institutional Review Board and conducted in accordance with the Declaration of Helsinki. The study was conducted at Florida Hospital, Orlando Campus. Patients who were admitted to the HF unit were screened and enrolled for the study. The inclusion criteria were patients admitted with decompensated HF with ejection fraction (EF) less than or equal to 40% and age between 20 and 89 years. Exclusion criteria were noncardiac disease with a life expectancy of less than one year, HF due to uncorrected valvular heart disease, psychiatric illness interfering with an appropriate follow-up, inability to understand study procedure, and inability to provide informed consent. Primary endpoint was 30-day readmission rate and the KCCQ score. Admission comorbid conditions, demographics, laboratory, echocardiographic data, and medications on discharge were secondary endpoints.

For every patient who met the study criteria, a trained research assistant explained the study to the patient and administered the KCCQ after a written informed consent was obtained. The assessment was generally completed within 1–3 days before discharge. A follow-up conversation was performed over the telephone 30 days after discharge to determine if rehospitalization occurred or not. Postdischarge readmission information was gathered through follow-up interview with the patient.

To evaluate associations between KCCQ score and readmission within 30 days after discharge, we first compared the difference between the nonreadmission group and readmission group in terms of the KCCQ scores, demographic characteristics, comorbidity, medications, and laboratory data using univariate analysis. In the univariate analysis,* t*-test was used for continuous variable, and Fisher's exact test was used for count number analysis. We then performed multivariate analysis to investigate how each clinical factor was associated with HF readmissions after controlling for the other factors. In the multivariate analysis, logistic regression models were used, and adjusted odds ratios (OR) were estimated for each factor hypothesized to predict HF readmission. We included HF readmission as a dependent variable and all potential factors as independent predictors in the logistic regression irrespective of whether they showed a significant difference between readmission and nonreadmission groups in the univariate analysis.

After the multivariate analysis, we further constructed five simplified prediction models and evaluated the importance of KCCQ score in the final model through comparing area under receiver operating characteristic curve (ROC) of each model. In this analysis, we also used integrated discrimination improvement (IDI), described by Pencina et al., to measure the average increase in model sensitivity penalized for average decrease in specificity with the addition of new variables [11]. In the prediction models, age was transformed to every 10-year increment, ejection fraction to every 10% decrease, KCCQ score to every 25-point increment, and sodium level to binary variable (<135 or ≥135).

Two hundred and twenty-eight (228, or 95%) patients had complete data for all variables. However, 12 (5%) patients had missing data in either age or race. As no nested missing pattern was detected, multiple imputation models were used for data imputation. As age was a continuous variable and race was a binary variable, normal linear regression was used for age while logistic regression was used for race imputation. All analyses were performed by Stata version 14 (StataCorp., 2015). All *P* values were two-tailed, and *α* < 0.05 was set as the level of statistical significance for all tests.

## 3. Results

In total, 240 patients were enrolled in the study. Forty-eight (20%) patients were readmitted within 30 days after discharge for HF while 192 (80%) patients were not readmitted or readmitted for reasons other than HF (Table 1). There was no significant difference between the nonreadmitted and readmitted patients in terms of average age (63.0 versus 59.9 yrs, *P* = 0.163), initial length of hospital stay (11.2 versus 9.7 days, *P* = 0.420), or percentage of white patients (59.9% versus 56.3%, *P* = 0.743). However, a significant difference between these two groups was noted on comparing gender, with male patients being more prone to being readmitted than female (85.4% versus 68.8% for male and 14.6% versus 31.3% for female, *P* = 0.020). None of the comorbidities showed significant difference in the relative frequency between the readmission and nonreadmission group (Table 1).

The KCCQ score, lab test results on admission, and discharge medications were compared between the nonreadmitted and readmitted patients (Table 2). The average KCCQ score was significantly higher in the nonreadmitted patients than in readmitted patients (40.8 versus 32.6, *P* = 0.019). Compared to readmitted patients, nonreadmitted patients had a higher ejection fraction on admission (24.7% versus 21.8%, *P* = 0.021). However, no significant difference was detected on comparing discharge medications, blood sodium level, or HGB between the two groups of patients in the univariate analysis (Table 2).

**Figure 1:**
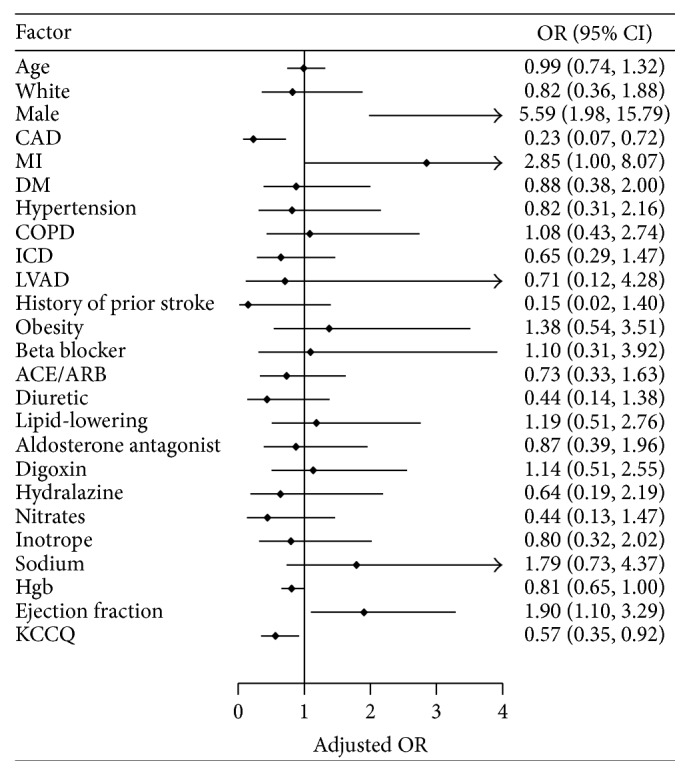
Adjusted odds ratios of readmission within 30 days after discharge derived from multivariate logistic regression analysis.

To further investigate the effect of each independent variable while controlling other covariates, multivariate analyses were performed (Table 3 and Figure 1). The results showed that the KCCQ score and EF were negatively associated with readmission rate (adjusted OR = 0.566 and 1.903 and *P* = 0.022 and 0.021, resp.) and that males were more likely to be readmitted than females (adjusted OR = 5.589, *P* = 0.001). Interestingly, patients with MI were more likely (adjusted OR = 2.849, *P* = 0.049) and patients with CAD were less likely to be readmitted (adjusted OR = 0.231, *P* = 0.012), compared to patients with other comorbidities. One possible interpretation could be that patients who have had a myocardial infarction are more likely to have wall motion abnormalities and fixed myocardial defects and thus a lower ejection fraction than those with nonobstructive coronary artery disease without an MI, leading to opposite contribution to HF readmission.

**Figure 2:**
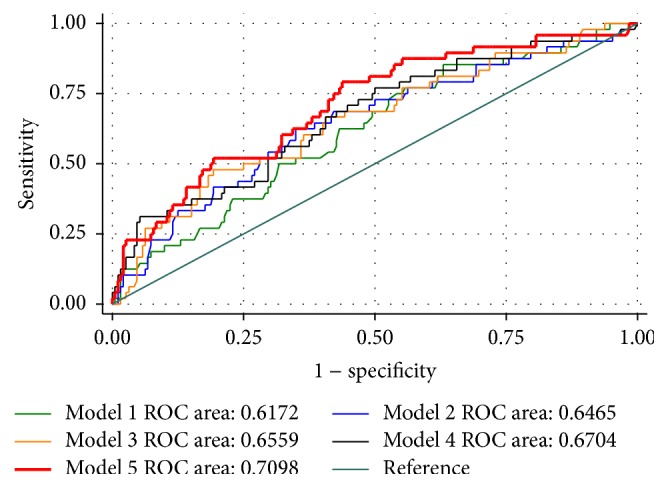
Comparison of ROC area among different models. Model 1: logit (read30) = age + gender; model 2: logit (read30) = age + gender + beta_blocker + ace/arb; model 3: logit (read30) = age + gender + beta_blocker + ace/arb + sodium + hgb; model 4: logit (read30) = age + gender + beta_blocker + ace/arb + sodium + hgb + ef; and model 5: logit (read30) = age + gender + beta_blocker + ace/arb + sodium + hgb + ef + KCCQ. read30: readmission in 30 days.

In order to evaluate how much contribution the KCCQ score made in predicting HF readmission, we developed a model by including seven factors besides KCCQ score (model 5) based on the multivariate regression results, published literature, and models. The* c*-statistic indicated that model 5 which included KCCQ score and all other potential predictors had the highest* c*-statistic value (0.710) among other reduced models without KCCQ score (Figure 2). As seen in Table 4, the IDI analysis demonstrated that the discriminatory performance of model 5 improved by 6.6% from the base model (model 1) that only included age and gender and by 2.7% from the reduced model (model 4) including all factors but the KCCQ score (this is the absolute increment; when compared with model 4, the IDI of the full model with KCCQ, model 5, increased by 2.7/3.9 = 69%). On the other hand, as an established independent factor associated with HF readmission [12, 13], EF increased the IDI from 1.3% (model 3) to 3.9% (model 4). These results suggested that the KCCQ score, as a single independent variable, is one of the important factors that could potentially be used for predicting readmission rates of HF patients within 30 days after discharge, and a combination of all these important factors would offer the greatest incremental gain.

## 4. Discussion

In this prospective study, we found that the KCCQ score was significantly associated with short-term HF readmission rate. It contributed to improving the* c*-statistics of a model based on age, gender, medications, laboratory data, and LVEF available at discharge from 0.670 to 0.710 and raised the IDI by 2.7%, which suggested that it may be helpful in predicting 30-day readmission and thus significantly improve prediction reliability when combined with other critical components. These findings may provide some help to guide follow-up strategies towards delivering optimal care, such as encouraging patients with lower KCCQ to have an early follow-up [14].

Lots of efforts have been made to identify the predictable factors that are associated with high risk of being readmitted, which has been quite challenging until now. In this study, we found that HF patients who had lower KCCQ score at time of discharge and lower EF and of male gender seemed to be more prone for readmission within 30 days. These findings were similar to some studies but not others. As a matter of fact, no specific patient or hospital factors have been shown to consistently predict 30-day readmission after hospitalization for HF. In a systematic review of 112 studies describing the association between traditional patient characteristics and readmission after hospitalization for HF, left ventricular EF, as well as other factors such as demographic characteristics, comorbid conditions, and New York Heart Association class, was associated with readmission in only a minority of cases [13]. In another meta-analysis of 69 studies and 144 factors for short-term readmission, noncardiovascular comorbidities, poor physical condition, history of admission, and failure to use evidence-based medication, rather than cardiovascular comorbidities, age, or gender, were more strongly associated with short-term readmission [15].

The KCCQ scores have been demonstrated to have much greater sensitivity to clinical changes in HF patients than the New York Heart Association (NYHA) functional classification, Minnesota Living with Heart Failure Questionnaire (LiHFe), and Short Form-36 (SF-36) [6]. The EVEREST trial suggested that the KCCQ is an important prognostic indicator of readmission within one year after discharge [9]. In their study, patients with KCCQ scores < 25 (worse health status) had more than threefold increased risk of the combined endpoint of rehospitalization and mortality than those in the best health status tier (KCCQ score > 75). More recently, KCCQ score was used to assess the feasibility of reflecting the changes of acute HF during hospitalization and predicting 30-day readmission. The authors found that it was feasible to use the KCCQ during acute HF hospitalizations and was sensitive to clinical improvement, but score changes during hospitalization did not predict 30-day readmission. However, this study was a relatively small study that included only 54 patients and was focused on KCCQ score differences during hospitalization between nonreadmission and admission groups [10]. In contrast, more than 240 patients were enrolled in our study and the KCCQ score was higher in nonreadmitted HF patients and was independently associated with lower 30-day readmission.

As mentioned above, there are multiple factors contributing to HF readmission; therefore, risk prediction models including and weighing all relevant factors were developed. In these models, discrimination, defined by the area under the receiver operating characteristic (ROC) curve, is used to tell how well a model can separate those who will have the outcome from those who will not have the outcome of interest. In this case, if the predicted risks for readmitted patients are all higher than for patients who are not readmitted, the model discriminates perfectly with* c*-statistic of 1. Conversely, if risk prediction is no better than chance, the* c*-statistic is 0.5. Models are typically considered reasonable when the* c*-statistic is greater than 0.7 and strong when the* c*-statistic is greater than 0.8 [16]. For 30-day readmission after HF hospitalization, several models have been developed. Only two models have generated* c*-statistics greater than 0.6 after studying both derivation and validation cohorts. One of them is the automated model developed by Amarasingham et al. incorporating data from the electronic health record at the time of hospitalization [17]. The other model combined claims-based demographic and comorbidity data with clinical data including vital signs, laboratory values, and measured left ventricular ejection fraction [18]. However, neither of the two models included KCCQ scores. Given only 48 readmissions in our study population, we included only 7 parameters besides the KCCQ score in the full model (model 5). Low EF and gender (men) resulting in increased odds ratios for readmission in the multivariate analysis were included; we also included information about medications, beta-blocker and ACE inhibitor/ARB, which had demonstrated lowering HF mortality [19–21], and sodium and Hgb, which may affect HF rehospitalization and mortality [22, 23] and have been used in other models (http://www.readmissionscore.org/heart_failure.php), although they were not independently associated with readmission in the multivariate analysis. The full model (model 5), which included the KCCQ score, increased the* c*-statistics of 0.617 in base model 1 based on age and gender to 0.710, with an IDI increase of 6.6%. Given that many other possible risk factors have not been included in this model, such as GFR and BNP, this model may not be perfect, although its* c*-statistics was greater than 0.7, and may exaggerate the contribution of the KCCQ score. However, our results suggested that the contribution of KCCQ for predicting short-term HF readmission could potentially be as important as LVEF.

The present findings should be considered within the context of the study's limitations. This study was performed in a single-community medical center, and further studies in other centers or multiple centers need to be done to validate our findings. We only administered the KCCQ one time during the hospitalization, which would not reflect changes between admission, during hospitalization, and after hospitalization. We did not collect some relevant medical history, such as history of admission due to heart failure in the past; physical examination findings; some other labs such as GFR and BNP, or chest X-ray findings. These factors could also be important in the risk prediction model.

## Figures and Tables

**Table 1:** Summary of demographic characteristics and medical history between HF readmission and nonreadmission within 30 days after discharge.

Demographic characteristics	Readmission within 30 days after discharge		
	No (*n* = 192)	Yes (*n* = 48)	*P* value
Age, yrs, mean (SD)	63.0 (13.6)	59.9 (14.5)	0.163
LOS, days, mean (SD)	11.2 (11.6)	9.7 (7.6)	0.420
*Race*			0.743
White	115 (59.9)	27 (56.3)	
Other	77 (40.1)	21 (43.8)	
*Gender*			0.020
Female	60 (31.3)	7 (14.6)	
Male	132 (68.8)	41 (85.4)	
*Comorbidity*			
CAD	137 (71.4)	28 (58.3)	0.085
MI	75 (39.1)	20 (41.7)	0.744
DM	103 (53.7)	26 (54.2)	1.000
Hypertension	156 (81.3)	38 (79.2)	0.838
COPD	44 (22.9)	10 (20.8)	0.848
ICD	100 (52.1)	22 (45.8)	0.519
LVAD	8 (4.2)	2 (4.2)	1.000
History of prior stroke	19 (9.9)	1 (2.1)	0.139
Obesity	52 (27.1)	13 (27.1)	1.000
At least one comorbidity	186 (96.9)	46 (95.8)	0.662

*Note*. Numbers in the parenthesis are percentage except indicated.

**Table 2:** Summary of KCCQ score, lab tests, and discharge medication between HF readmission and nonreadmission within 30 days after discharge.

Demographic characteristics	Readmission within 30 days after discharge		
	No (*n* = 192)	Yes (*n* = 48)	*P* value
KCCQ score, mean (SD)	40.8 (22.2)	32.6 (18.5)	0.019
*Lab on admission*			
Sodium, mean (SD)	137.6 (4.7)	137.5 (5.6)	0.915
HGB, mean (SD)	12.1 (2.1)	11.9 (2.1)	0.622
Ejection fraction	24.7 (7.4)	21.8 (8.8)	0.021
*Discharge medication*			
Beta blocker	172 (89.6)	43 (89.6)	1.000
ACE/ARB	110 (57.3)	25 (52.1)	0.520
Diuretic	168 (87.5)	40 (83.3)	0.478
Lipid-lowering	126 (65.6)	29 (60.4)	0.504
Aldosterone antagonist	98 (51.0)	23 (47.9)	0.748
Digoxin	60 (31.3)	15 (31.3)	1.000
Hydralazine	30 (15.6)	5 (10.4)	0.494
Nitrates	39 (20.3)	6 (12.5)	0.301
Inotrope	46 (24.0)	13 (27.1)	0.708

*Note*. Numbers in the parenthesis are percentage except indicated.

**Table 3:** Summary of multivariate analysis investigating the effects of demographic characteristics, medical history, discharge medication, lab test, and overall KCCQ score on readmission rate within 30 days after discharge (*n* = 240).

Factor	Adjusted OR	SE	95% CI	*P* value
Age	0.990	0.145	0.742–1.320	0.946
White	0.821	0.348	0.358–1.884	0.642
Male	5.589	2.962	1.979–15.79	0.001
CAD	0.231	0.135	0.074–0.724	0.012
MI	2.849	1.514	1.005–8.074	0.049
DM	0.877	0.369	0.384–2.001	0.754
Hypertension	0.815	0.405	0.308–2.157	0.681
COPD	1.084	0.514	0.429–2.744	0.864
ICD	0.648	0.271	0.286–1.471	0.299
LVAD	0.710	0.650	0.118–4.275	0.709
History of prior stroke	0.150	0.171	0.016–1.402	0.096
Obesity	1.377	0.658	0.540–3.511	0.503
Beta blocker	1.096	0.713	0.306–3.920	0.888
ACE/ARB	0.734	0.299	0.331–1.629	0.447
Diuretic	0.438	0.257	0.138–1.384	0.159
Lipid-lowering	1.186	0.511	0.509–2.761	0.693
Aldosterone antagonist	0.873	0.360	0.389–1.957	0.741
Digoxin	1.137	0.47	0.506–2.554	0.756
Hydralazine	0.639	0.402	0.186–2.193	0.476
Nitrates	0.443	0.271	0.134–1.467	0.182
Inotrope	0.799	0.378	0.316–2.022	0.636
Sodium	1.791	0.815	0.734–4.368	0.200
Hgb	0.810	0.087	0.655–1.000	0.050
Ejection fraction	1.903	0.532	1.100–3.292	0.021
KCCQ	0.566	0.141	0.347–0.922	0.022

**Table 4:** Prognostic value of readmission within 30 days after discharge of different models comparing to model 1 with only demographic predictors.

Model	*c*-statistics	IDI increase (%)	*P* value
Model 1: age + gender	0.617	—	—
Model 2: age + gender + beta_blocker + ace/arb	0.647	0.9	0.123
Model 3: age + gender + beta_blocker + ace/arb + sodium + hgb	0.656	1.3	0.081
Model 4: age + gender + beta_blocker + ace/arb + sodium + hgb + ef	0.670	3.9	0.005
Model 5: age + gender + beta_blocker + ace/arb + sodium + hgb + ef + KCCQ	0.710	6.6	<0.001

## Abbreviations

KCCQ: Kansas City Cardiomyopathy Questionnaire
HF: Heart failure
HRQL: Health-related quality of life
EF: Ejection fraction
LVEF: Left ventricular ejection fraction
OR: Odds ratios
CAD: Coronary artery disease
MI: Myocardial infarction
DM: Diabetes mellitus
COPD: Chronic obstructive pulmonary disease
ICD: Implantable cardioverter-defibrillator
LVAD: Left ventricular assist device
ACE: Angiotensin converting enzyme
ARBs: Angiotensin receptor blockers
HGB: Hemoglobin
IDI: Integrated discrimination improvement
NYHA: New York Heart Association
ROC: Receiver operating characteristic
GFR: Glomerular filtration rate
BNP: Brain natriuretic peptide.

## Additional Points

*Competency in Medical Knowledge*. Heart failure is one of the most common diagnoses associated with readmission. KCCQ score provided important prognostic information for predicting 30-day readmission and it can significantly improve prediction reliability along with other critical components.* Translational Outlook*. Additional clinical studies need to be done in multiple centers with a larger sample size to validate our finding. Future research should include relevant physical examination findings and chest X-ray findings, which could be important in the risk prediction model.
